# Application effect study of a combination of TeamSTEPPS with modularization teaching in the context of clinical instruction in trauma care

**DOI:** 10.1038/s41598-024-55509-4

**Published:** 2024-02-27

**Authors:** Tieying Qiu, Min Chen, Suyuan Gao, Jin Huang, Weixing Wang, Liping Wang, Haiyang Li

**Affiliations:** Clinical Nursing Teaching and Research Section, The Second Xiangya Hospital of Central South University, Changsha, 410011 China

**Keywords:** Modularization, TeamSTEPPS, Professional self-concept, Professional benefit, Health care, Patient education

## Abstract

To explore the effect of a combination of Team Strategies and Tools to Enhance Performance and Patient Safety (TeamSTEPPS) with modularization teaching in the context of clinical instruction in trauma care. A total of 244 nursing students who participated in clinical practice in orthopaedic wards from March 2020 to April 2022 were divided into two groups that received the same trauma care teaching content. The control group (n = 119) used the traditional teaching approach, and the experimental group (n = 125) utilized a combination of TeamSTEPPS with a modularization teaching model. A questionnaire was used to assess students’ theoretical knowledge, practical skills, self-concepts and professional benefits after one month with the goal of determining their end-of-course performance. The theoretical knowledge scores obtained by the control group and the experimental group were 89.56 ± 4.06 and 91.62 ± 2.84, respectively, and these results were statistically significant (P < 0.05). Students preferred the combination of TeamSTEPPS with the modularization teaching model to the traditional instructional method in terms of practical skills, professional self-concepts and professional benefits (P < 0.05). The application of the combination of TeamSTEPPS with modularization teaching in the context of clinical instruction in trauma care made significant contributions to nursing students’ mastery of theoretical knowledge and practical skills, enhanced their sense level of professional identity, instilled a correct occupational ideology in such students, and enhanced the professional benefits they were able to obtain.

## Introduction

Trauma is a common public health problem that is associated with tremendous social and economic burdens and results in 5.7 million annual deaths globally^1^. Trauma is the leading cause of death among young adults, which mostly include traffic injuries, fall injuries, crush injuries, and sharp instrument injuries^2^. Trauma accounts for approximately 7% of the human mortality rate worldwide, ranking fourth among the causes of death^3^. Specifically, the mortality rates associated with trauma are approximately 37.63/100,000 in urban areas and 53.49/100,000 in rural areas in China^4^. Therefore, trauma has come to represent a serious challenge for the Chinese healthcare system.

However, trauma care services in China are not ideal, especially at grassroots-level centres; for example, trauma care services in district- or provincial-level hospitals vary across different districts. Trauma care teaching requires nursing students to be capable of continuously evaluating, assessing, treating and reevaluating patients. Accurate judgement and correct treatment in short time periods are pivotal for saving patients' lives^5^. Trauma patients, who are mostly admitted to orthopaedics and spinal surgery departments, are associated with various clinical difficulties, such as complicated nursing tasks and tasks that require high levels of professional nursing skills. Traditional methods of nursing education, in which context students passively receive information imparted by teachers, limit the development of students' clinical thinking^6^. It is difficult to mobilize students’ enthusiasm if the traditional instructional approach is the only approach used in clinical practice. Some knowledge associated with instruction in trauma nursing cannot be adapted to changes in the emerging economy and epidemiologic transition^7^. In this context, China trauma care training (CTCT) is beginning to be implemented as a standardized training course for medical nurses, including through lectures, videos, conferences and workshops^8^. Nurses with different educational backgrounds may exhibit different levels of understanding of the skills instilled by such training^9^. However, only 1.7% of nurses in district or provincial-level hospitals have received specialty trauma training. Therefore, trauma care training is a crucial aspect of clinical teaching with regard to enhancing the skills of nurses in various emergency situations.

Modularization is an integrated approach to the task of dividing knowledge into its modular and clinically important aspects with the goals of stimulating students’ interest and providing a comprehensive overview of clinical knowledge^10^. The application of “modularization” in clinical teaching combines various types of knowledge and skills to improve students’ mastery of knowledge and skills by encouraging them to learn the content of the modules^11^. Modular teaching has been successfully applied to various medical subjects^12–14^. In addition, Team Strategies and Tools to Enhance Performance and Patient Safety (TeamSTEPPS), an approach which was developed by the American Department of Defense Patient Safety Program and the Agency for Healthcare Research and Quality, is an multifaceted, evidence-based instructional toolkit designed to promote teamwork and collaboration in healthcare delivery teams with the goal of improving patient safety and care quality^15,16^. By enhancing communication and teamwork among medical team members, TeamSTEPPS helps nurses respond quickly and effectively to various emergencies^17,18^. We introduced modular teaching and TeamSTEPPS training to provide essential knowledge with an adequate emphasis on fundamentals through a wide variety of learning activities. Therefore, we designed theoretical courses for student interns based on the characteristics of orthopaedic clinical teaching; these courses combined modular training with the TeamSTEPPS method. The goal of this research is to explore the effect of applying this combined approach in clinical nurse courses.

## Materials and methods

### Study design

We conducted a controlled trial from March 2020 to April 2022 in Hunan Province, China. Nurses involved in orthopaedic internships were recruited as the sample for this study. Four orthopaedics wards in two representative 3A hospitals (Xiangya Hospital and Second Xiangya Hospital of Central South University) were chosen for this research.

For both groups of students, the same chapter, “Trauma Care”, was used as the teaching content. All teaching processes for the experimental group and the control group were implemented during the same period. The Ethics Committee of the Second Xiangya Hospital of Central South University approved this study (No: E202071), and all methods were performed in accordance with the relevant guidelines and regulations of Second Xiangya Hospital. All the participants consented to participate in this research and signed informed consent forms after being fully informed of the details of this study. In this study, participants' personal information was anonymized, and these data were used only for this study.

### Participants

A total of 250 nursing students who practised in the orthopaedic department of our hospital were selected as the research subjects. Among these subjects, four students took more than one week off during the period in which this study was conducted, and two students ended their internship ahead of schedule; hence, a total of 244 students were involved in this research.

The inclusion criteria focused on full-time nursing students who had completed professional courses and participated in clinical practice, students who had finished one month of rotation in the orthopaedic department, and students who had been informed of and consented to participate in this study. The exclusion criterion focused on students who took a leave of absence for more than one week.

To ensure uniformity in terms of teaching methods, according to the participants’ sequence of admission, nursing students who practised in the orthopaedic department from March 2020 to February 2021 were assigned to the control group (n = 119), and those who practised in related departments from March 2021 to April 2022 were assigned to the experimental group (n = 125). The differences between the two groups in terms of gender, age, educational background, place of origin, status as only children, and duration of clinical practice were not statistically significant (P > 0.05), as shown in Table 1.

**Table 1:** Comparison between the experimental and control groups in terms of general information.

Basic information		Control group (n = 119)	Experimental group (n = 125)	χ^2^/t value	*P value*
Age		20.66 ± 0.682	20.53 ± 0.655	1.490^a^	0.138
Gender	Male	21	18	0.479^b^	0.489
	Female	98	107		
Educational background	Bachelor’s degree	25	24	0.124^b^	0.724
	Associate degree	94	101		
Place of birth	Countryside	75	79	0.001^b^	0.982
	Towns and villages	23	24		
	City	21	22		
Only child status	Yes	24	19	1.036^b^	0.309
	No	95	106		
Duration of clinical practice	≤ 3 months	23	40	1.545^b^	0.462
	4–6 months	32	36		
	≥ 7 months	55	76		

^a^t test; ^b^chi-square test.

### Teaching methods

#### Methods used for the control group

Traditional teaching methods were used for the control group. Student interns were presented with the department’s working environment, rules and regulations as well as requirements for interns as part of the introduction provided by teachers in the first week of practice.

Teachers first explained the relevant theoretical knowledge concerning the selected disease according to the syllabus’s specific requirements once per week. Subsequently, teachers guided students in observing the clinical process, practicing and answering clinical questions. Finally, the teachers summarized the course content and evaluated the students’ levels of comprehension.

#### Hybrid modularization and the TeamSTEPPS teaching model

##### Curriculum framework design

At the beginning of the curriculum formulation process, teachers used the “Surgical Nursing” and “Nursing for Emergency Treatment” published by People's Medical Publishing House as teaching references. This course module was designed based on content pertaining to trauma first aid in the context of orthopaedics as well as content related to spinal surgery. According to the knowledge mastery methods exhibited by the nursing students, the instructions were divided into three modules, i.e., the basic quality module, the post skill module and the comprehensive skill module, as shown in Table 2. These three modules were implemented over a period of four weeks, and each module was predefined in terms of its time, objective, content and form.

**Table 2:** Instructional framework, modules and contents.

Modules	Time	Objective	Content	Form
Basic quality	Week 1	Learn basic professional theories and master necessary knowledge points concerning operations to lay the foundation for the two subsequent modules	Theories, training in basic quality	Course
Post skill	Weeks 2–3	Combine theories and practice to improve professional skills	Representative trauma first aid cases in orthopaedics for actual group practice	Group discussion
Comprehensive skill	Week 4	Improve capabilities with regard to analysis, problem-solving, comprehensive management and coordination	Simulated case to support field practice, operations in a real- life scenario	Simulation practice

**Figure 1:**
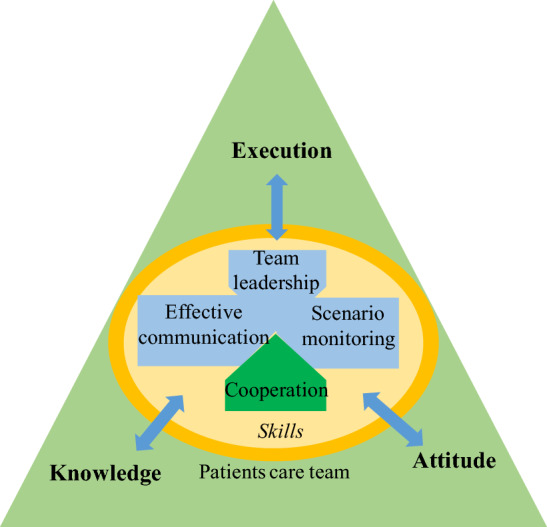
Teaching mode associated with the TeamSTEPPS approach.

Meanwhile, beginning during the second week of the internship, the TeamSTEPPS model was integrated into the course framework, which included three elements—team building, execution and attitude—and 4 skills—team leadership, effective communication, situation monitoring and cooperation. The contents of the TeamSTEPPS method were incorporated into the post skill and comprehensive skill modules and were specifically assessed, as shown in Fig. 1.

##### Curriculum implementation

Several course modules were used to implement the TeamSTEPPS for the nursing students who participated in this study, and the details are presented below.

1. Basic quality module: Before participating in the internship, students could download teaching resources from the WeChat group to complete the preassessment. During the first week of the study, the students were provided with theoretical knowledge and basic quality training for eight hours on the first and fifth days of the first week, respectively. In line with the syllabus, teachers listed the specific requirements concerning theoretical knowledge and clinical skills related to trauma cases, such as the classification and definition of trauma care.
2. Post skill module: During the second week, the students participated in all the activities in a group format. Several representative clinical trauma first aid cases were chosen for actual practice. The following four major skill modules were provided, as presented in Table 3: (1) ventilation first aid; (2) cardiopulmonary resuscitation; (3) circulatory first aid; and (4) trauma first aid. The students combined the teacher's demonstrations of operations with the theoretical knowledge they had learned, which they then practised by themselves for an hour in each module. One skill included in each module was selected randomly for examination, and all groups were asked to provide comments on and score each other’s results.
3. Comprehensive skill module: Five cases were included in this module: (1) a car accident scene; (2) a fire scene; (3) a high-altitude falling scene; (4) an earthquake scene; and (5) a traffic collision scene. Every group selected one student to serve as the simulated patient, and the remaining group members designed the specific situation in accordance with the scenario they had randomly selected in advance. The students first assessed the injury and used relevant equipment to treat the patient. In addition, teachers assessed their performance in terms of teamwork, problem-solving capability and professional knowledge and skills.

**Table 3:** Contents of the post skill module.

Module	Contents	Methods	Week/duration
Ventilation first aid	Manually open airway (airway obstruction), open (closed) thoracic injury, balloon-assisted ventilation	Observation operations Simulation operation	Second week/1 h
Cardiopulmonary resuscitation	Manual CPR, use of a defibrillator	Observation operations Simulation operation	Second week/1 h
Circulatory first aid	Liquid resuscitation in haemorrhagic shock, indwelling needle transfusion, monitoring of vital signs	Observation operations Group discussion	Third week/1 h
Trauma first aid	Method for dealing with spinal cord fracture patients, fixation method for limb fracture patients, triage and recognition of different injuries	Presentation of the case Group discussion	Third week/1 h

### Data collection

Before the formal research was conducted, eight teachers who oversaw two groups were selected as investigators. The two teachers were recruited from the same ward. The inclusion criteria for these teachers were as follows: (1) at least five years of work experience in the orthopaedics or spinal department, (2) willingness to participate in this study, and (3) experience conducting scientific research and good communication skills. These eight teachers selected investigators to participate in face-to-face training, which lasted for two days, and they recognized the purpose, method, and research process of this study, which was conducted at the Second Xiangya Hospital of Central South University. Only after completing the training and receiving the relevant qualification could these teachers initiate the research at their hospitals.

### Assessed variables

To evaluate the effect of the combination of modularization with the TeamSTEPPS teaching model in the context of trauma care, at the end of the internship, students were required to complete theoretical examinations and practical skills assessments. Moreover, an anonymous questionnaire administered via the online platform; this questionnaire included the Professional Self-Concept of Nurses Instrument and Nurses Perceived Professional Benefit Scale, and it was used to assess students’ communication skills, teamwork spirit, professional identity and teaching satisfaction. The details of the variables thus assessed are presented below.

Theoretical examination and practical skills assessment: The total score for theoretical and practical skills examination was 100. With regard to the theoretical exam, questions were selected randomly according to the syllabus, distributed to the students via the Wenjuanxin platform (http://www.wjx.cn), and then scored by the teachers. The practical skills examination was divided into five parts (trauma assessment, treatment proficiency, accuracy of aid skill, team cooperation and problem-solving capability), and scores of 20 scores for each sector. The higher the score is, the better the student’s practical skills.

In the Chinese version of the Professional Self-Concept of Nurses Instrument (PSCNI-CV), Nurses’ self-concepts are associated with various important aspects, including the development of clinical competencies, quality of care, job satisfaction and professional commitment^19^. The PSCNI was developed by Dr. Arthur of Hong Kong Polytechnic University in 1995^20^. The scale consists of 30 items across 5 dimensions: leadership, flexibility, professional skill, satisfaction and communication capability. This measure is scored on a four-point Likert-type scale ranging from 1 = “disagree” to 4 = “agree” (24 items) or from 1 = “agree” to 4 = “disagree” (6 items). Total scores on the PSCNI-CV range from 30 to 120 points. The Cronbach’s α of this measure was 0.871, and the reliability was 0.7.

Nurses Perceived Professional Benefit Scale (NPPB): NPPB focuses on positive emotional experiences in the context of nursing practice. Nurses can perceive the benefits associated with their profession during the course of nursing practice and can agree that the nursing profession is able to promote their overall growth based on the theory of positive psychology^21^. The NPPB scale was developed by Hu Jing^22^ and includes 33 items across 5 dimensions: personal growth, good nurse‒patient relationships, recognition from families and friends, positive professional perceptions and a sense of belonging to a work team. This measure is scored on five-point Likert-type scale ranging from 1 = “completely disagree” to 5 = “completely agree”. Total scores range from 29 to 145 points. The Cronbach’s α was 0.958. The subscale coefficients ranged from 0.821 to 0.893.

### Statistical analyses

All the statistical analyses were conducted using SPSS software (ver. 22.0; IBM Corp, Armonk, NY, USA). Continuous data are presented in terms of the mean ± standard deviation (x ± s), and categorical variables are presented in terms of frequencies (%). The χ^2^ test was used to evaluate the survival rates of the two groups. Significance was assessed using an independent sample t test. Values of P < 0.05 were considered to indicate statistical significance.

## Results

### Differences between the experimental and control groups in terms of theoretical knowledge and practical skill

**Figure 2:**
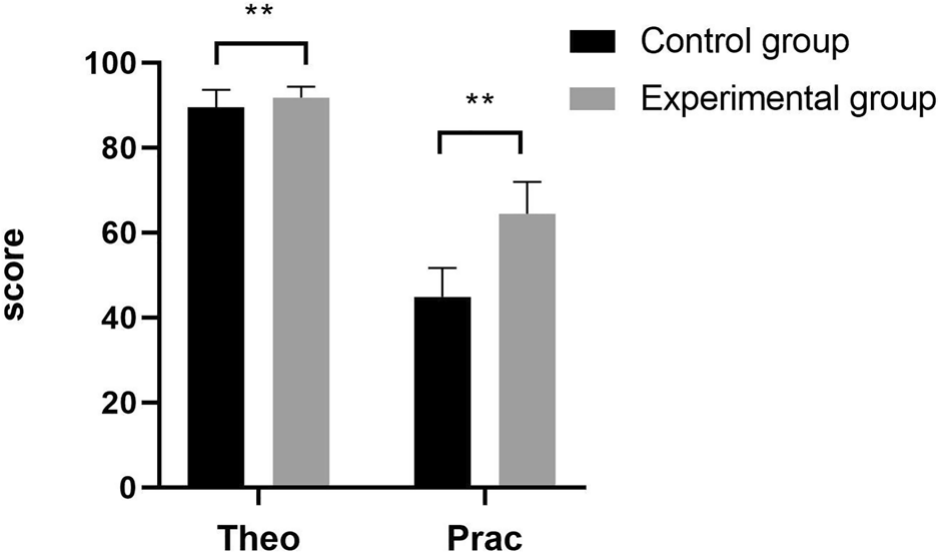
Comparison between the experimental and control groups in terms of the theoretical examination and practical skills. **: P < 0.001; Theo: Theoretical examination; Prac: Practical skill.

To assess the effect of the combination of modularization with the TeamSTEPPS teaching model, we compared the theoretical examination scores attained by the two groups. The theoretical examination score attained by the control group was 89.56 ± 4.06, and that attained by the test group was 91.62 ± 2.84; p values less than 0.05 indicated a significant difference. As shown in Fig. 2 and Table 4, the theoretical knowledge and practical skills of the students in the experimental group were significantly greater than those of the students in the control group. In terms of practical skills, trauma care teaching using the combination of modularization with the TeamSTEPPS teaching model was proven to improve the mastery of knowledge among participants (P < 0.001).

**Table 4:** Comparison between the experimental and control groups in terms of the of theoretical examination and practical skill assessment.

Variable	Control group (n = 119)	Experimental group (n = 125)	t	P
Theoretical examination	89.56 ± 4.06	91.62 ± 2.84	− 4.608	0.001
Practical skill	44.73 ± 7.00	63.75 ± 8.08	− 19.62	< 0.001
Trauma assessment	8.08 ± 4.91	15.86 ± 2.39	− 15.847	< 0.001
Treatment proficiency	8.97 ± 2.27	11.88 ± 3.05	− 8.433	0.003
Accuracy of aid skill	8.84 ± 2.39	11.38 ± 3.03	− 7.237	0.037
Team cooperation	8.10 ± 2.88	13.80 ± 4.18	− 12.356	0.001
Problem-solving capability	10.74 ± 2.05	10.84 ± 1.81	− 0.407	0.685

### Comparison between the experimental and control groups in terms of the PSCNI-CV

**Figure 3:**
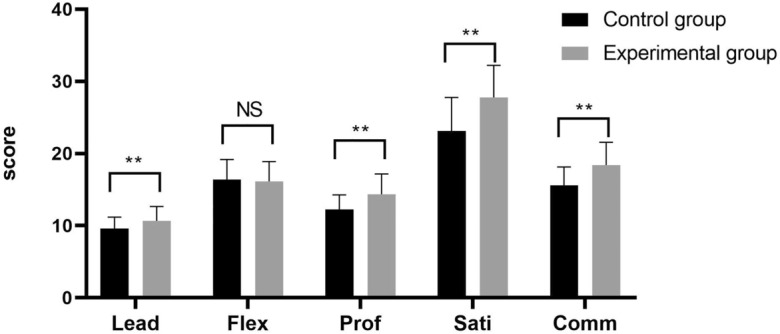
Comparison between the experimental and control groups in terms of the PSCNI-CV. **: P < 0.001; NS: not statistically significant; Lead: leadership; Flex: flexibility; Prof: professional Skills; Sati: satisfaction; Comm: communication capability.

The PSCNI-CV is an instrument used to assess professional self-concepts, which represent a future strength of nurses. With regard to the PSCNI-CV, the experimental groups exhibited more positive professional self-concepts (87.37 ± 9.62) than did the control groups (76.92 ± 9.35) (Table 5), as indicated by higher scores in terms of leadership, professional skills, satisfaction and communication (P < 0.05) (Fig. 3).

**Table 5:** Comparison between the experimental and control groups in terms of the PSCNI-CV.

Variable	Control group (n = 119)	Experimental group (n = 125)	t	P
Total score on the PSCNI-CV	76.92 ± 9.35	87.37 ± 9.62	− 8.598	< 0.001
Leadership	9.61 ± 1.59	10.69 ± 1.98	− 4.66	< 0.001
Flexibility	16.40 ± 2.79	16.13 ± 2.76	0.775	0.439
Professional skills	12.23 ± 2.04	14.36 ± 2.81	− 6.754	< 0.000
Satisfaction	23.13 ± 4.64	27.78 ± 4.44	− 8.022	< 0.000
Communication capability	15.55 ± 2.60	18.42 ± 3.14	− 7.729	< 0.000

### Comparison between the experimental and control groups in terms of the NPPB

**Figure 4:**
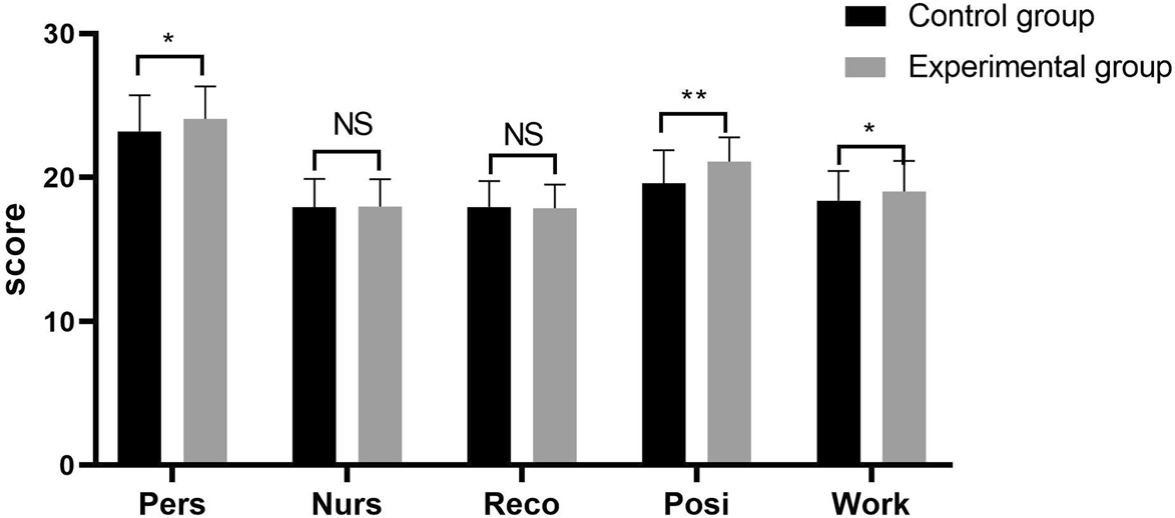
Comparison between the experimental and control groups in terms of the NPPB scale. **P < 0.001; *P < 0.05; NS: not statistically significant; Pers: personal growth; Nurs: good nurse-patient relationships; Reco: recognition from families and friends; Posi: positive professional perceptions; Work: sense of belonging to a work team.

Nurses can obtain more benefits from the nursing profession if they are willing to continue to grow and develop within the profession. The differences in NPPB scores between the experimental and control groups are presented in Table 6. The experimental group exhibited higher scores than the control group with regard to three aspects of the NPPB scale, namely, personal growth, positive professional perceptions and sense of belonging to a work team (P < 0.05). No significant differences were observed with regard to the aspects of good nurse‒patient relationships or recognition from families and friends (P > 0.05) (Fig. 4).

**Table 6:** Comparison between the experimental and control groups in terms of the NPPB.

Variable	Control group (n = 119)	Experimental group (n = 125)	t	P
Total NPPB scores	97.03 ± 6.03	100.00 ± 4.80	− 4.261	< 0.001
Personal growth	23.19 ± 2.52	24.06 ± 2.25	− 2.845	0.005
Good nurse–patient relationships	17.92 ± 1.97	17.96 ± 1.90	− 0.176	0.860
Recognition from families and friends	17.94 ± 1.81	17.85 ± 1.66	0.420	0.676
Positive professional perceptions	19.60 ± 2.29	21.10 ± 1.69	− 5.843	< 0.001
Sense of belonging to a work team	18.38 ± 2.07	19.02 ± 2.12	− 2.408	0.017

## Discussion

Trauma care is a professional skill that requires cooperation among multiple subjects. Teamwork skills are essential to the task of ensuring the quality of care and patient safety, thereby facilitating effective patient care and preventing adverse events^23^. Nurses are important members of interprofessional teams and within-nursing-care teams^24^. However, in the context of in-hospital triage in the Chinese context, multidisciplinary treatment and divisional treatment cannot adapt to the typical features of emergencies, such as suddenness, criticality and high mortality rates^25,26^, thus making it difficult to secure optimum treatment for patients. Hence, it is critical to improve the trauma care capability and teamwork skills of medical students.

Hitherto, several innovations in teaching for nurse students in Chinese medical universities and hospitals have been identified, such as the flipped classroom, PBL (problem-solving learning), the massive online open course (MOOC) model and scenario simulation teaching^27–30^. Although the creative teaching methods mentioned above have enriched traditional courses greatly and improved the practical skills of nurse students, they have ignored the need to cultivate teamwork ability. In this study, the teaching model consisted of three modules, namely, basic quality, post skill and comprehensive skill, alongside the TeamSTEPPS method. In the basic quality module, students could acquire various aspects of theoretical knowledge, such as the definition and classification of trauma. In the post skill module and the comprehensive skill module, students could practice and solve problems pertaining to certain clinical trauma cases with the goal of improving their learning motivation and cooperation; they could subsequently improve their practice skills in the simulation model. The results of this research revealed significant, positive changes in the experimental group in terms of theoretical knowledge and practical skills. The TeamSTEPPS model is a student-centred teaching method and observation system that has been adopted by many colleges and universities^31,32^. The results of the new teaching method were consistent with the results of this study with regard to stimulating students’ learning interest and enthusiasm and improving their teaching efficiency. Students may assess their learning process and feedback by using a combination of modularization with the TeamSTEPPS teaching method, which is conducive to the tasks of summarizing the content and enhancing problem-solving capabilities in clinical practice.

Professional self-concept, which is based on the individual’s personal attitudes toward his or her occupation, professional responsibility, ideals and values, represents the core of nurses’ occupational choices and development^33^. This study revealed that differences in the dimensions of such a professional self-concept, such as management ability, professional skills, satisfaction and communication capability, were statistically significant (P < 0.05). Students who obtained higher scores with regard to their professional self-concepts exhibit superior abilities with regard to the acquisition of knowledge and skills. Professional self-concepts are correspondingly enhanced when students’ independent thinking, flexibility and professional skills are improved through scenario simulation learning^34^. The possession of a positive professional self-concept is a protective and efficient factor against burnout, and this capability can prevent emotional exhaustion^35^. Education and the acquisition of knowledge impact nurses’ job satisfaction and self-concept^36^. Nurses who exhibit stronger professional self-concepts are more accountable with regard to their patients and work results, and they can provide care to patients with more respect and interest^37^. Figure 3 shows that little difference was observed between the control group and the experimental group in terms of the flexibility of students. We speculated that the combination of modularization with the TeamSTEPPS teaching method can emphasize the task of changing students’ behaviour. The students were able to address unexpected problems in teamwork, improve their professional skills, and develop accurate professional self-concepts. Students who received the combination of modularization with the TeamSTEPPS teaching method could not only acquire theoretical knowledge but also cultivate positive professional self-concepts, thus promoting their empowerment, clinical performance, and problem-solving ability and subsequently enhancing the quality of nursing^38^.

Professional benefit is a positive occupational emotion. Good professional benefits constitute an internal driving force for professional development. In this study, the perceived professional benefits perceived by nursing students were at the upper-middle level, thus leaving a great deal of room for improvement. The subscale for personal growth was associated with the highest scores, thus clearly indicating that nurses can perceive benefits as a result of personal growth. Through constant learning and accumulation, nurses’ professional knowledge and skills can be improved, which could benefit the professional development and growth of nurses themselves^39^. Several studies have shown that practicability, participation and initiative are indispensable for promoting nurses’ learning interests^40^. A combination of TeamSTEPPS with modularization teaching represents a more flexible model for motivating students’ independence and sensitivity, thus effectively cultivating clinical assessment and treatment^41^. Therefore, good perceived professional benefits can help mitigate nurses’ negative emotions, increase their subjective well-being, stimulate their innovative behaviours, improve their work efficacy and reduce their rates of job burnout.

## Conclusion

Participatory learning involving high adaptability and operability can fully mobilize students' independent learning abilities and enable them to develop basic operational skills, practical skills and creative capabilities in the context of clinical practice^42^. A combination of TeamSTEPPS with modularization teaching can optimize instruction resources, which has the strongest effect on students. This new teaching method is an innovative approach that combines a modular model with the TeamSTEPPS model, thereby increasing the accuracy of theoretical knowledge and enhancing professional self-concepts and professional benefits. This study nevertheless has certain limitations. First, the students included in this study were recruited from the same city, i.e., Hunan, China. Further rigorous studies featuring large sample sizes and a multicentre focus are necessary. Second, this study divided the sample groups in accordance with the sequence of admission rather than obtaining a random sample. The main reason for this approach was to avoid interference between two simultaneous groups, which would bias the results of our overall and subgroup analyses. Further studies could use different methods, such as by conducting qualitative research or employing different grouping methods, to collect more data and increase the rigor of the study design. Third, the participants in this study were nursing students, who represent the main actors in trauma care. Future studies may use the TeamSTEPPS model to investigate various subjects with different majors to explore significant differences in this context.

## Data Availability

The datasets used and(or) analyzed during the current study available from the corresponding author on reasonable request.
